# Supplementary material for: Efficiency of Interferon-γ in Activating Dendritic Cells and Its Potential Synergy with Toll-like Receptor Agonists
Source: Viruses. 2023 May 19;15(5):1198. doi: 10.3390/v15051198 (PMC10224459; doi:10.3390/v15051198)
Supplement: Supplementary file 1 [file viruses-15-01198-s001.zip › viruses-2370336-supplementary.pdf]

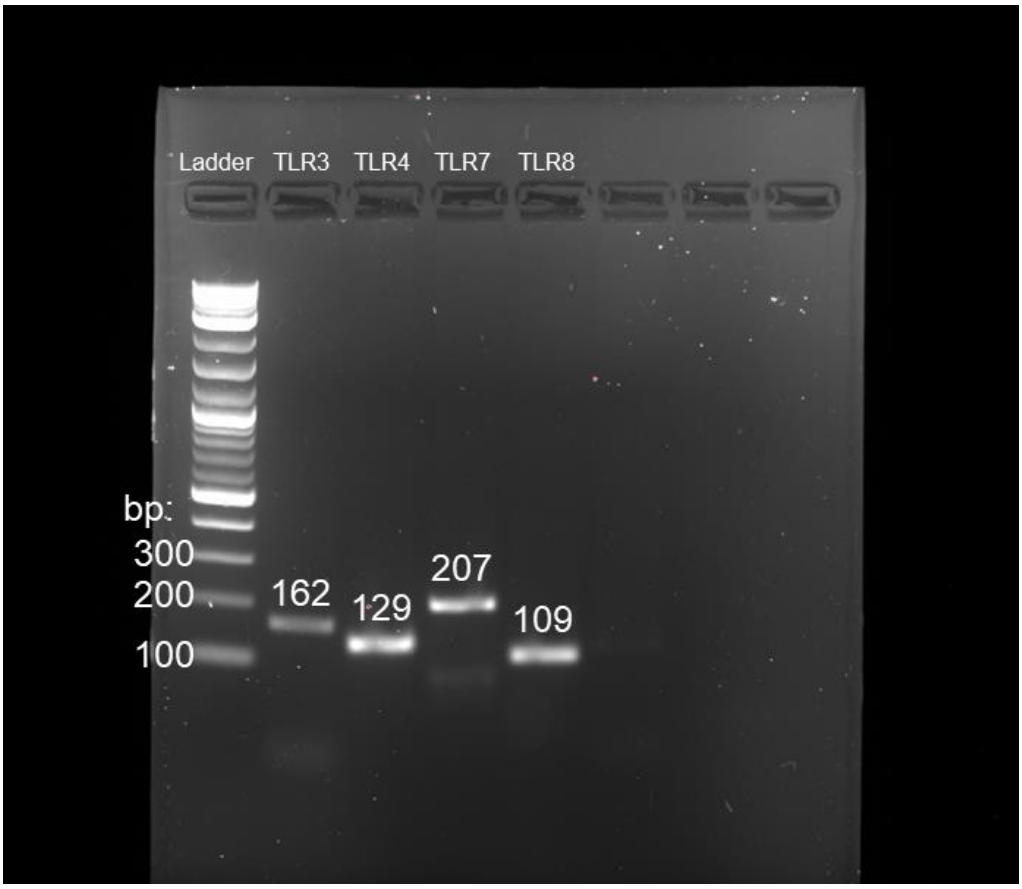

Supplemenmtary Figure S1. Agarose gel (2.0%) electrophoresis of the PCR products from the cDNA of JAWSII murine dendritic cells. Ladder: DNA marker NEB N3200; TLR3: the tlr3 PCR primer product of 162 bp; TLR4: the tlr4 PCR primer product of 129 bp; TLR7: the tlr7 PCR primer product of 207 bp; TLR8: the tlr8 PCR primer product of 109 bp.
